# Accurately tracking single-cell movement trajectories in microfluidic cell sorting devices

**DOI:** 10.1371/journal.pone.0192463

**Published:** 2018-02-07

**Authors:** Jenny Jeong, Nicholas J. Frohberg, Enlu Zhou, Todd Sulchek, Peng Qiu

**Affiliations:** 1 Department of Biomedical Engineering, Georgia Institute of Technology and Emory University, Atlanta, Georgia, United States of America; 2 School of Electrical and Computer Engineering, Georgia Institute of Technology, Atlanta, Georgia, United States of America; 3 School of Industrial and System Engineering, Georgia Institute of Technology, Atlanta, Georgia, United States of America; 4 School of Mechanical Engineering, Georgia Institute of Technology, Atlanta, Georgia, United States of America; Johns Hopkins University, UNITED STATES

## Abstract

Microfluidics are routinely used to study cellular properties, including the efficient quantification of single-cell biomechanics and label-free cell sorting based on the biomechanical properties, such as elasticity, viscosity, stiffness, and adhesion. Both quantification and sorting applications require optimal design of the microfluidic devices and mathematical modeling of the interactions between cells, fluid, and the channel of the device. As a first step toward building such a mathematical model, we collected video recordings of cells moving through a ridged microfluidic channel designed to compress and redirect cells according to cell biomechanics. We developed an efficient algorithm that automatically and accurately tracked the cell trajectories in the recordings. We tested the algorithm on recordings of cells with different stiffness, and showed the correlation between cell stiffness and the tracked trajectories. Moreover, the tracking algorithm successfully picked up subtle differences of cell motion when passing through consecutive ridges. The algorithm for accurately tracking cell trajectories paves the way for future efforts of modeling the flow, forces, and dynamics of cell properties in microfluidics applications.

## Introduction

**Fig 1:**
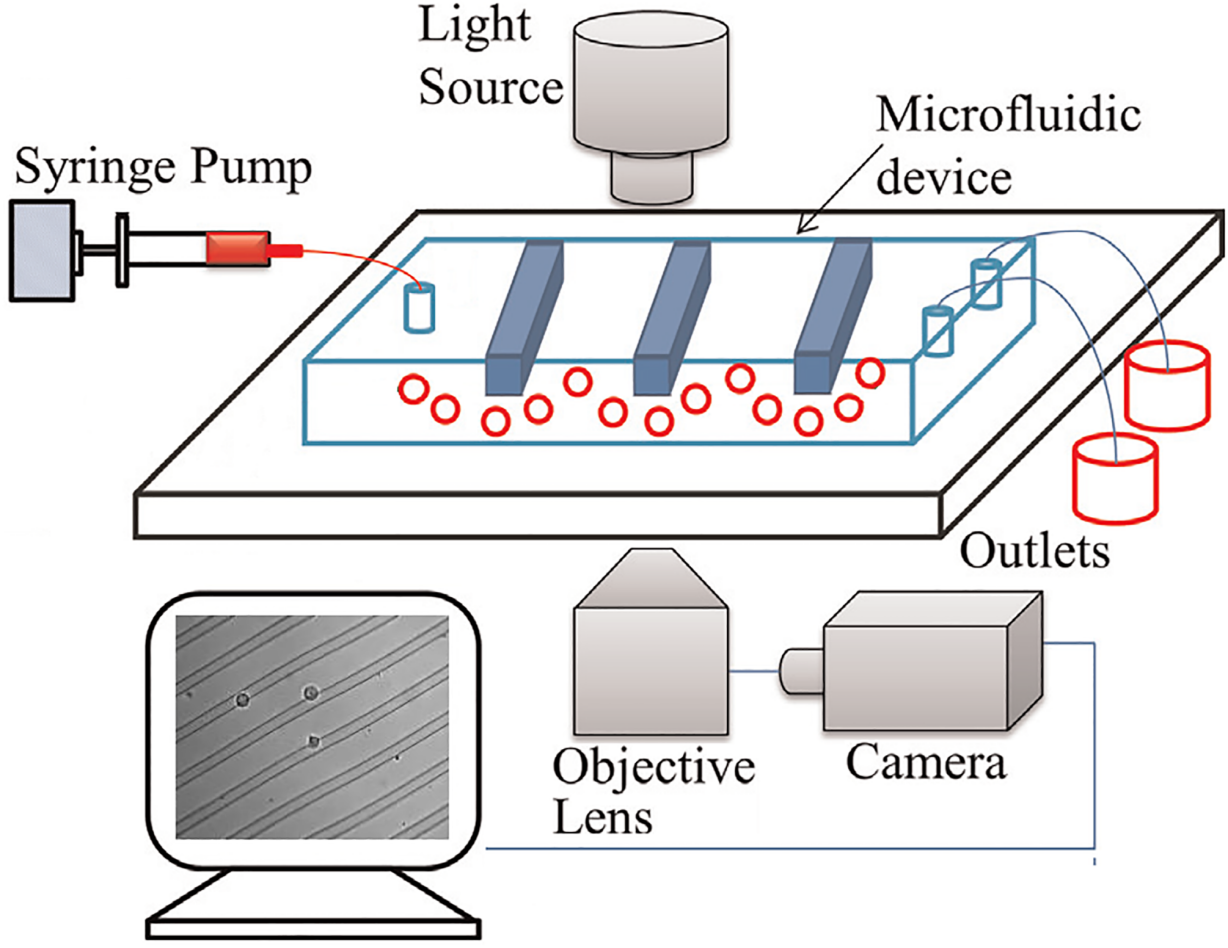
Cartoon illustration of a ridged microfluidic channel. A system that can be used for sorting cells according to their biomechanical properties.

Microfluidics is a promising technology for biological inquiries at the single-cell level, such as single-cell gene expression for lineage analysis [1, 2] and signaling dynamics [3], microfluidic cell sorting [4]. One interesting application is the study of single-cell biomechanical characteristics, such as elasticity, viscosity, stiffness and adhesion [5]. Using a microfluidic channel decorated with ridges that are diagonal with respect to the flow direction (Fig 1), cells are compressed and translated when passing through the channel, and exhibit different trajectories depending on their biomechanical properties. The trajectories are also affected by the channel design, in terms of the ridge height, angle, and spacing. The microfluidic approach for studying cellular biomechanics is highly cost effective compared to atomic force microscopy, and has high throughput similar to flow cytometry. Ridged microfluidic channels have been used to separate cells based on stiffness [6], size [7], adhesion [8, 9], viability [10], and viscoelasticity [11].

**Fig 2:**
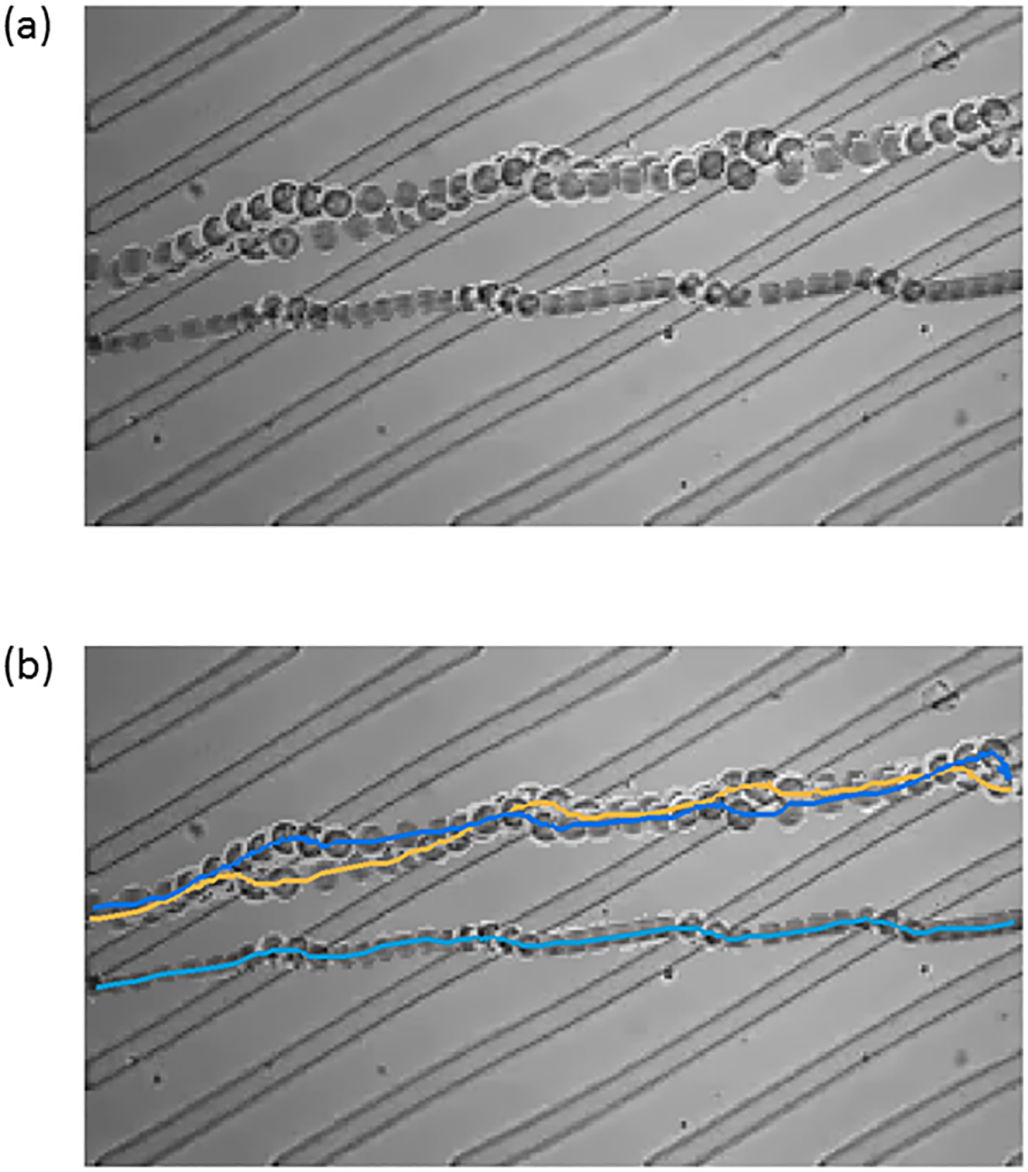
Example data. (a) a short segment of video recording shown by overlapping multiple frames, (b) desired single-cell trajectories to be extracted.

The trajectories contain rich information pertaining to the interactions between the cells and the ridged channel, providing an opportunity for quantifying cell biomechanical properties, as well as optimizing the channel design for various sorting applications. By mounting the microfluidic chip on an inverted microscope and a high-speed camera, cells can be recorded when passing through the channel, and the trajectories can be computationally extracted from the recordings. Fig 2a shows an example cell trajectory by overlaying multiple frames of a recording.

In this application, extracting the trajectories from the recordings appears to be a simple problem, because cells can be easily segmented from the relatively constant background. In addition, cells do not divide, do not significantly change their shapes, and move toward the same general direction. However, it is still challenging to automatically extract the trajectories with high accuracy. Depending on the experimental setup, multiple cells can pass through the channel at the same time, traveling at varying speed due to their biomechanical properties. Some cells may even get trapped by the ridges. We observed many examples where one cell catches up and collides with another, and the two cells stick together for a while before detaching from each other. The collision and detachment of cells makes it challenging to accurately segment cells in each frame, and calls for joint considerations of consecutive frames. We have explored several existing automated computational tools for cell tracking and particle tracking, including MosaicSuite in ImageJ [7, 12], CellProfiler [13], CellTrack [14] and TLA [15]. However, all of them had difficulties in automatically and accurately tracking the trajectories of cells in this application, due to either the collision and detachment, contrast patterns in the background, or cells with drastically varying speed.

In this paper, we develop a computational pipeline for automatically extracting single-cell trajectories from video recordings of cells moving through ridged microfluidic channels. The pipeline contains three steps: frame-by-frame foreground identification and segmentation, forward matching between consecutive frames, and backward matching between consecutive frames. Using this pipeline, cell trajectories can be extracted with high accuracy. Although the initial segmentation step does not properly separate cells touching each other, the forward and backward matching steps address this issue. Even if two cells stick together when entering and exiting the video, as long as they are ever separated in any frame in between, our pipeline is able to correctly identify their single-cell trajectories.

## Materials and methods

### Experimental setup

To demonstrate microfluidic sorting and cell tracking algorithm, we fabricated a ridged microfluidic channel using standard replica molding [6], and examined K562 lymphoblastic cells in the ridged microfluidic channel. The K562 cells were purchased from ATCC. K562 cells were cultured at 37 Celsius and 5% CO2 in RPMI 1640 media, supplemented with 10% FBS and 1% pen/strep. K562 cells were chemically treated using actin depolymerizing agent cytochalasin D (CD) for 2 hours of incubation at a 1.5 uM concentration to create cells that differed by their stiffness but were substantially of similar size. Untreated K562 cells (0CD) were stiff, whereas the 1.5 *μM* CD treated K562 cells (1.5CD) were softened. The stiff and soft K562 cells were separately prepared in a cell flow buffer of 28 mL PBS and prepared to minimize cell adhesion (12 mL Percoll with 40 mg BSA and 1.6 mg EDTA added) to be at 1 million cells per mL concentration and flowed through the microfluidic channel at a 0.05 mL/min flow rate. The experiments were recorded using an inverted microscope (Nikon Eclipse Ti) using the 20X magnification objective and videos recorded using a high-speed camera (Phantom v7.3 Vision Research using PCC 2.6 Phantom Camera Software). The camera was placed such that the field of view captured the area around the center of the microfluidic channel, but did not cover the entire channel. In order to accurately record the cell trajectories, we operated the high-speed camera at a minimum of 800 frames per second with a minimum resolution of 640 by 480 pixels.

### Cell tracking algorithm

The trajectories of cells passing through microfluidic sorting devices can be recorded by a high-speed camera. Our goal is to automatically extract the trajectories from the gray-scale video recordings. For the example data in Fig 2a, the desired trajectories are shown in Fig 2b. Although time is not shown explicitly, the two cells with entangling trajectories passed through the channel together, and they collided and detached a few times. One cell caught up and collided with the other, the doublet rotated, and the follower became the leader when the two cells detached. This happened again later and the order of the two cells switched back. The video clip corresponding to this example is included in S1 File. This example highlights one challenge in this analysis, how to automatically handle the collision and detachment of cells. Our computational pipeline contains three steps: frame-by-frame foreground identification and segmentation, forward matching between consecutive frames, and backward matching between consecutive frames. We implemented the tracking algorithm in Matlab. Source code and example use cases are available in S1 File.

### Foreground identification and segmentation

In our experimental setup, the microfluidic device and the camera are both fixed. As a result, the background stays relatively constant, without any rotation, translation or deformation. The baseline intensity of the background varies, due to slight changes in the illumination condition during the experiments. Therefore, the background of each frame can be estimated by the median of nearby frames.

**Fig 3:**
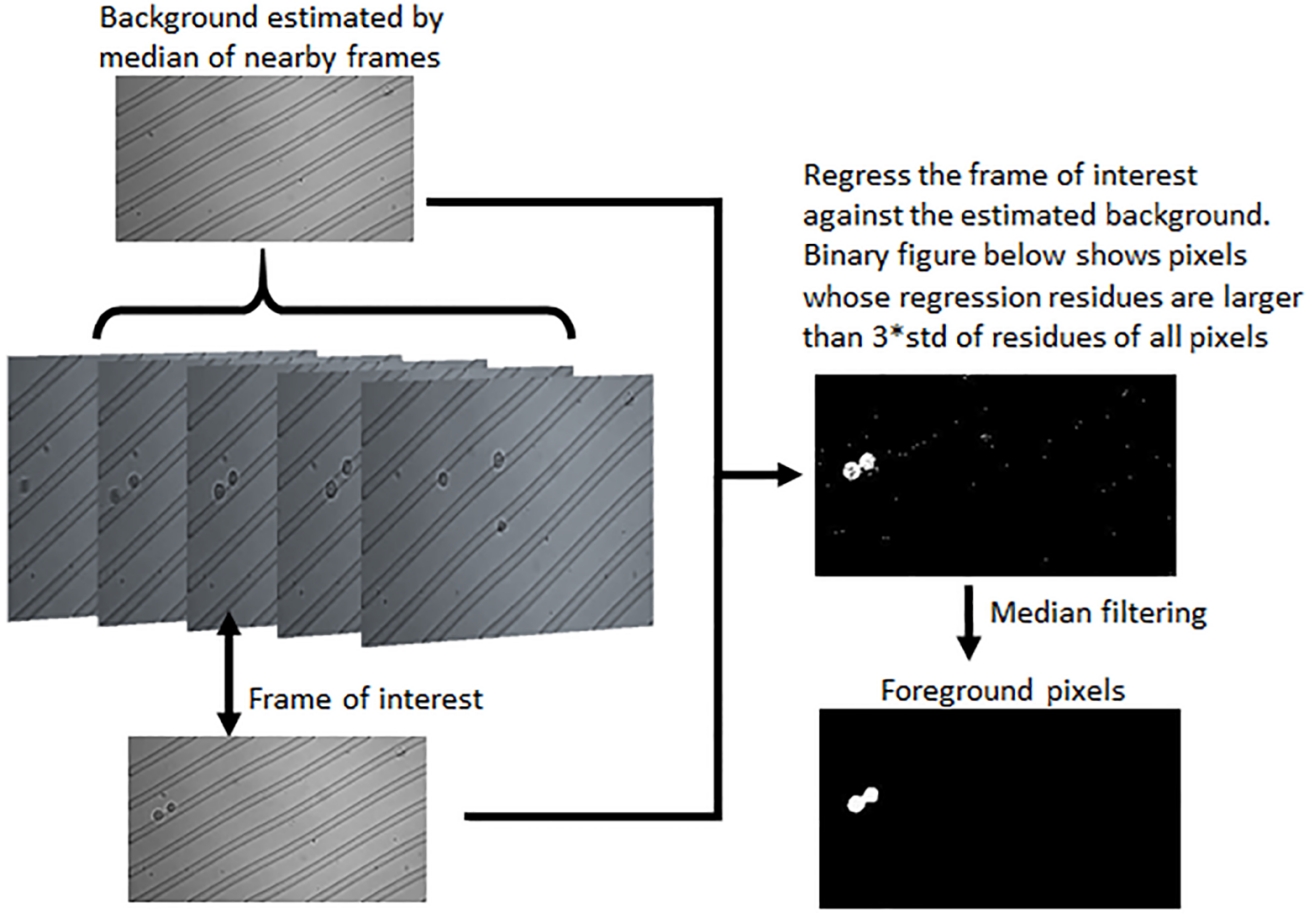
Foreground identification. We first estimate the background by the median of nearby frames, then perform linear regression of the frame of interest against the estimated background, threshold the regression residue to identify foreground pixels, and finally perform median filtering to refine the foreground.

To identify the foreground objects in an image frame, we first estimate the background by taking the median of nearby frames within a small window (default is 300), as shown in the illustrative example in Fig 3. We perform a linear regression to predict the image frame by the estimated background. The prediction residues for all the pixels are fitted by a Gaussian distribution. The foreground pixels can be identified by the collection of pixels with residues larger than three standard deviation away from the mean of the residues of all pixels. The foreground is further refined by median filtering to remove noise, and filling in the holes to recover low contrast pixels in the cell nucleus. The foreground pixels can be visualized as a binary image in the bottom-right of Fig 3. We then perform segmentation on the binary representation of the foreground, by computing its connected components. We call each component an *event* in the image frame, which may correspond to a cell, an aggregate of multiple cells, a piece of debris, or noise. In this particular example in Fig 3, the foreground consists of only one event, which is an aggregate of two cells.

The outputs of this preprocessing step in the pipeline are the events extracted from each frame. For each event, we compute the (*x*, *y*) position of its center, the number of pixels, the radius defined by the maximum distance from the pixels to the center, the mean and standard deviation of the pixel intensities. The cell ID of each event is initialized as NaN, meaning that these events are not associated with any cells yet. The subsequent forward and backward matching steps will segment the aggregates and associate these events to cells.

The appropriate window size for estimating background depends on the speed of the slow moving cells. If the window size is so small that a slow moving cell appears to be stagnant, it will not be identified as the foreground. Our choice of window size is 300, which corresponds to 0.1 seconds in real time. We have tested a wide range of this parameter from 100 to 500, and observed that the algorithm is not sensitive to this parameter. This algorithm can produce incorrect foreground if a cell is temporarily stuck in the microfluidic channel, moving extremely slowly for > 150 consecutive frames (half of the window size), which rarely happen in our data. The median filtering window size is 5-by-5, which is effective in removing noise in foreground identification. It also removes events smaller than 4 pixels in size, which are typically debris and not of interest.

### Forward matching of consecutive frames

Each cell passing through the channel should appear in a series of consecutive frames, and should ideally produce one event in each of those frames. This may not be true due to various reasons. For example, a cell with low contrast in one frame may not be detected as an event in the frame-by-frame foreground identification step; a cell can generate multiple events in one frame if part of it is of low contrast which leads to over segmentation; an aggregate of multiple cells in one frame only produces one event. We develop a forward matching algorithm to compare an image frame to its immediate subsequent frame, associate the events to cells, and handle the above situations by merging or segmenting the events when necessary. The algorithm generates all possible matchings of events between the two consecutive frames, scores the possible matchings, and applies the one with the highest score.

**(1) Generate all possible sets of matchings of events between the two frames.** From the current frame to the next one, each event in the current frame can either disappear, or match to a nearby event in the next frame whose position is further along the flow direction. Similarly, each event in next frame can either suddenly appear, or match to a nearby event upstream of the flow direction in the current frame. Events appear or disappear when cells enter or exit the channel. Two events are considered “nearby” if the distance between their centers is within 5 times of the maximum of their radii. This parameter defines the speed limit of cells that can be tracked. For an extremely fast-moving cell whose center position is far away in the current and next frames, it will be considered as two cells, one disappearing after the current frame and another appearing in the next frame. However, our data does not contain such fast-moving cells, which is guaranteed by the sampling rate of the high-speed camera and the flow rate in the microfluidic channel in our experimental setup.

**Fig 4:**
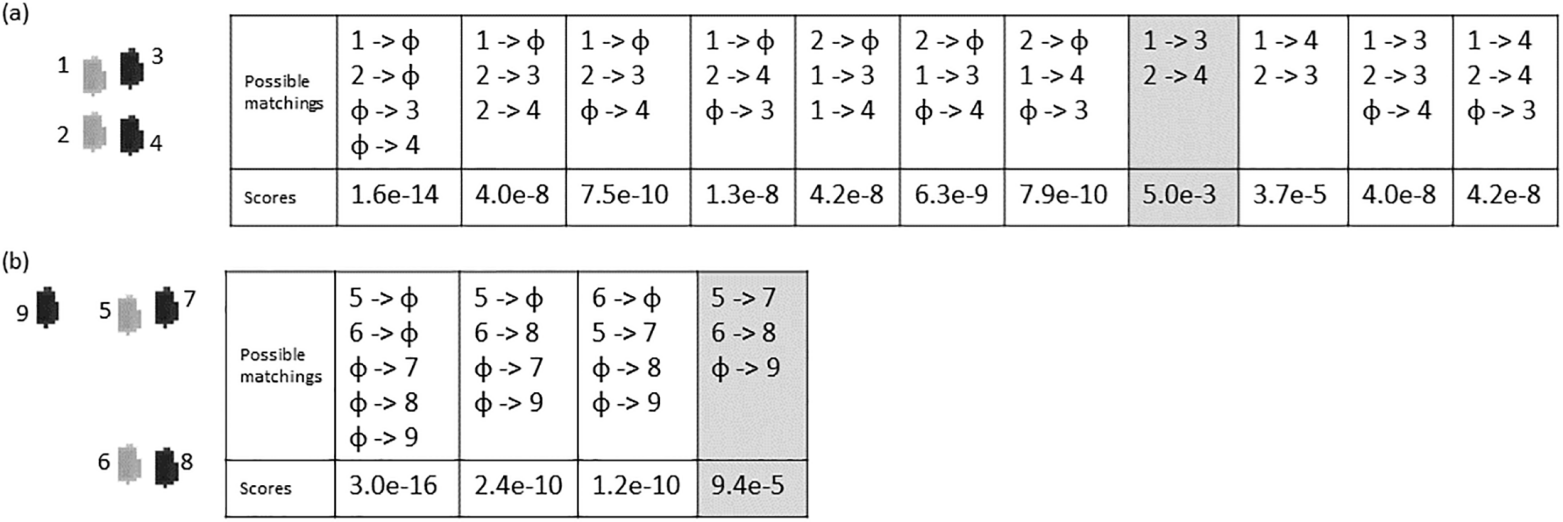
Matching events in consecutive frames. Two examples of sets of possible matchings between events in current frame (light gray) and events in the next frame (dark gray), along with scores of the matchings. Numbers are used to label the events.

We use *ϕ* to denote an empty set. A *ϕ*-to-one matching represents an event that suddenly appears in the next frame and does not match to any event in the current frame. A one-to-*ϕ* matching represents an event in the current frame disappears in the next frame and does not match to any event in the next frame. In addition to *ϕ*-to-one (appearing) and one-to-*ϕ* (disappearing), the matchings can also be one-to-one, one-to-multiple or multiple-to-one. A one-to-multiple matching represents an aggregate becoming detached cells, and a multiple-to-one matching represents multiple cells colliding and forming an aggregate. We do not consider matchings that are multiple-to-multiple. Fig 4 shows two examples. In Fig 4a, compared to events 1 and 2 in the current frame, events 3 and 4 in the next frame are nearby and further downstream the flow direction (left to right). Therefore, both events in the next frame can potentially match to both events in the current frame, allowing a total of eleven possible sets of matchings. In Fig 4b, the total number of possible sets of matchings is four, smaller than the previous case, because not all events in the next frames are downstream and nearby all events in the current frame. Event 9 in the next frame is upstream of all events in the current frame, meaning that it must have just appeared. Although event 8 in the next frame is downstream of both 5 and 6 in the current frame, it can only match to event 6 because it is far away from event 5.

**(2) Score the possible sets of matchings.** Since cells do not divide or significantly change their shape when moving through the channel, for two events that correspond to the same cell in two consecutive frames, their center positions, sizes and pixel intensities should be similar. We use differences in these aspects to score matching events, and derive an overall score for each possible set of matchings.

Let *M* denote a particular set of matchings, which may contain one-to-*ϕ*, *ϕ*-to-one, one-to-one, one-to-multiple, and multiple-to-one matchings. For an event *i*, denote its size (number of pixels) by *P*_*i*_, its center position (*x*_*i*_, *y*_*i*_), mean and standard deviation of pixel intensities *μ*_*i*_ and *σ*_*i*_. For a collection of multiple events *j*_1_, …*j*_*k*_, denote their overall mean and standard deviation of pixel intensities as *μ*_(*j*_1_, …, *j*_*k*_)_ and *σ*_(*j*_1_, …, *j*_*k*_)_. For each individual matching in this set, we score each type of matching as follows:

$$S\left(i\to \phi \right)={\left(\frac{1}{P_{i}}\right)}^{2}$$ (1)

$$S\left(\phi \to j\right)={\left(\frac{1}{P_{j}}\right)}^{2}$$ (2)

$$S\left(i\to j\right)={\left(\frac{1}{P_{i}-P_{j}}\right)}^{2}\left(\frac{1}{\left|x_{i}-x_{j}||y_{i}-y_{j}\right|}\right)\left(1-\frac{\mu_{i}-\mu_{j}}{\text{max}\left(\mu_{i},\mu_{j}\right)}\right)\left(1-\frac{\sigma_{i}-\sigma_{j}}{\text{max}\left(\sigma_{i},\sigma_{j}\right)}\right)$$ (3)

$$\begin{array}{lll} S\left(i\to j_{1},\ldots ,i\to j_{k}\right) & = & {\left(\frac{1}{P_{i}-\sum_{k}P_{j_{k}}}\right)}^{2}\left(\frac{1}{{\text{max}}_{k}(|x_{i}-x_{j_{k}}|){\text{max}}_{k}(|y_{i}-y_{j_{k}}|)}\right)\cdots \\ & & \cdots \left(1-\frac{\mu_{i}-\mu_{\left(j_{1},\ldots ,j_{k}\right)}}{\text{max}\left(\mu_{i},\mu_{\left(j_{1},\ldots ,j_{k}\right)}\right)}\right)\left(1-\frac{\sigma_{i}-\sigma_{\left(j_{1},\ldots ,j_{k}\right)}}{\text{max}\left(\sigma_{i},\sigma_{\left(j_{1},\ldots ,j_{k}\right)}\right)}\right) \end{array}$$ (4)

$$\begin{array}{lll} S\left(i_{1}\to j,\ldots ,i_{k}\to j\right) & = & {\left(\frac{1}{\sum_{k}P_{i_{k}}-P_{j}}\right)}^{2}\left(\frac{1}{{\text{max}}_{k}(|x_{i_{k}}-x_{j}|){\text{max}}_{k}(|y_{i_{k}}-y_{j}|)}\right)\cdots \\ & & \cdots \left(1-\frac{\mu_{\left(i_{1},\ldots ,i_{k}\right)}-\mu_{j}}{\text{max}\left(\mu_{\left(i_{1},\ldots ,i_{k}\right)},\mu_{j}\right)}\right)\left(1-\frac{\sigma_{\left(i_{1},\ldots ,i_{k}\right)}-\sigma_{j}}{\text{max}\left(\sigma_{\left(i_{1},\ldots ,i_{k}\right)},\sigma_{j}\right)}\right) \end{array}$$ (5)

The overall score of a set of matchings is defined as the product of scores of individual matchings in the set. The basic idea here is to compare the matched events, and penalize changes in size, distance of movement, and differences in pixel intensity distribution.

The illustrative examples shown in Fig 4 are constructed by replicating one cell extracted from our data. Therefore, all events in these two examples are exactly 53 pixels in size, and share the same pixel intensity distribution. Distances between nearby events range from 10 to 15 pixels. Fig 4 shows the overall scores of all possible sets of matchings, where we can see that the most reasonable set receives the highest score in both examples.

**(3) Apply the best set of matchings.** With the scoring function to identify the best set of matchings between events in two consecutive frames, we develop an algorithm to perform forward matching of consecutive frames, associating events to cells. To initialize the algorithm, each event in the first frame is considered as a different cell, and assigned with a unique cell ID. After that, based on the best set of matchings between events in the first and second frames, the algorithm determines which events in the second frame are associated to cells in the first frame, and which events in the second frame are new cells that should be assigned with new cell IDs. Iteratively, the algorithm examines the second and third frames, the third and fourth frames, and continues until the last two frames.

Given the cell IDs of events in frame *f*, and the best set of matchings between frames *f* and *f* + 1, we apply the matchings using the algorithm detailed in Table 1. For one-to-*ϕ* matchings that represent events disappearing, nothing needs to be done. For *ϕ*-to-one matchings of events appearing, the newly appeared events in frame *f* + 1 are assigned with new cell IDs. For a one-to-one matching, the event in frame *f* + 1 is associated to the cell ID of the matching event in frame *f*.

**Table 1:** Algorithm for forward matching of consecutive frames.

1: Each event in the first frame is assigned a unique cell ID. 2: **for***f* = 1: number of frames—1 **do** 3: Compare the events from frame *f* to frame *f* + 1, generate all possible sets of matchings and score them. 4: Identify the best set of matching with highest score. 5: **for** each matching in the best set **do** 6: **if** matching represent event disappearing (*i* → *ϕ*) **then** 7: Do nothing. 8: **end if** 9: **if** matching represent event appearing (*ϕ* → *j*) **then** 10: Assign a new cell ID to event *j*. 11: **end if** 12: **if** one-to-one matching (*i* → *j*) **then** 13: Assign the cell ID of event *i* to event *j*. 14: **end if** 15: **if** multiple-to-one matching (*i*_1_ → *j*, …, *i*_*k*_ → *j*) **then** 16: Compute pairwise distance for *i*_1_, …, *i*_*k*_ based on the associated cells in frames 1 ∼ *f*. 17: **while** (The smallest pairwise distance < radius of event *j*) **do** 18: Merge the two corresponding events, and assign a new cell ID to the merged event 19: Recompute the pairwise distance in based on the corresponding cells in frames 1 ∼ *f*. 20: **end while** 11: **if** (Events *i*_1_, …, *i*_*k*_ are merged to one event *i*) **then** 22: Assign the cell ID of event *i* to merged event *j*. 23: **else** 24: Use the remaining events in frame *f* as templates to split event *j* into multiple events. 25: Assign the cell ID of each remaining event in frame *f* to the events generated by splitting *j*. 26: **end if** 27: **end if** 28: **if** one-to-multiple matching (*i* → *j*_1_, …, *i* → *j*_*k*_) **then** 29: Compute pairwise distance for events *j*_1_, …, *j*_*k*_ in frame *f* + 1. 30: **while** (The smallest pairwise distance < radius of event *i*) **do** 31: Merge the two corresponding events. 32: Recompute pairwise distance. 33: **end while** 34: **if** (Events *j*_1_, …, *j*_*k*_ are merged to one event *j*) **then** 35: Assign the cell ID of event i to merged event *j*. 36: **else** 37: Assign new and unique cell IDs to each remaining event after the merge. 38: **end if** 39: **end if** 40: **end for** 41: **end for**

**Fig 5:**
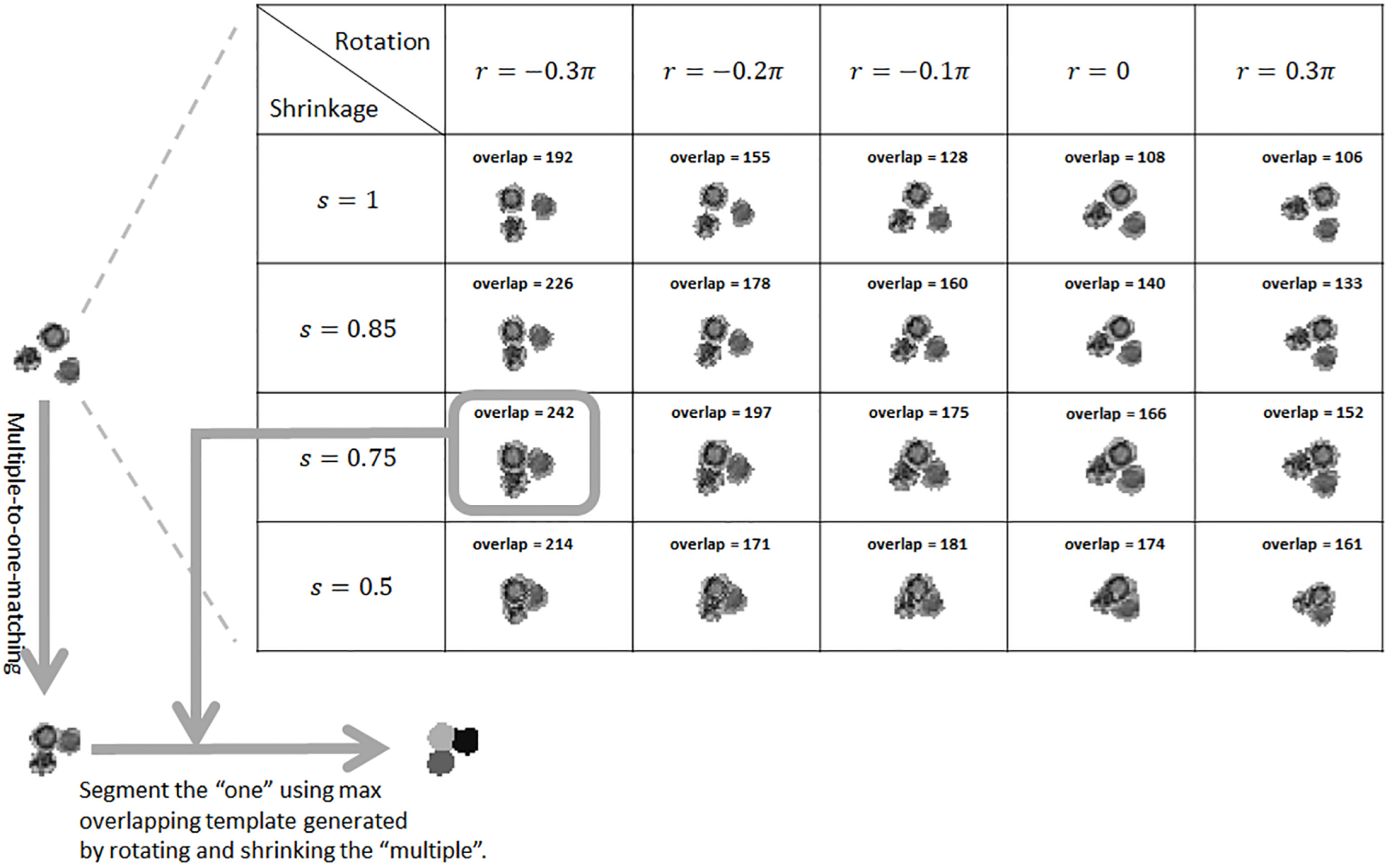
Multiple-to-one matching. By shrinking the distances among the multiple events and rotating them together, templates are generated to represent possible configurations of the multiple in the next frame. Templates are evaluated by their number of pixels that overlap with the one event in the next frame. The maximal overlapping template is used to segment the one event in the next frame into multiple pieces, turning the multiple-to-one matching into one-to-one.

For a multiple-to-one matching, we first examine the cell IDs of the multiple events in frame *f*. For each pair of these cells, we identify all previous frames where they co-exist, compute the distance between their centers in each of those frames, and compute the maximum distance. Doing this for each pair of cells in the “multiple” forms a pairwise matrix of maximum distances, which represents evidence of whether these “multiple” events in frame *f* have been separated in previous frames. Since these events in frame *f* match to one event in frame *f* + 1, we define a threshold using the radius of the “one” event. If the smallest element of this pairwise matrix is smaller than the threshold, meaning the two corresponding events have never shown decent separation in the frames 1 ∼ *f* analyzed so far, we merge them into one event and assign to it a new cell ID. We then re-compute the pairwise matrix for the remaining events of the “multiple”, and check whether any pair should be merged. This is essentially an agglomerative clustering process that merges events in the “multiple” that have never been decently separated. If all the events in the “multiple” are merged together, the matching reduces to the one-to-one case. If not, it is still a multiple-to-one situation. We use the multiple events in frame *f* to construct templates to segment the matched one event in frame *f* + 1. The templates are constructed by shrinking the distances among the multiple events by a varying scalar (*s* < = 1) and rotating them by a varying angle (*θ* ∈ [−*π*, *π*)). Using the template that overlaps most with the “one” event, we segment the “one” event into multiple events, each of which is assigned with the cell ID of the corresponding event in frame *f*. Fig 5 shows an example of how a three-to-one matching is performed.

For a one-to-multiple matching, we also first examine the pairwise distances between the multiple events. Since they have not yet been associated to any cells, their pairwise distances can only be computed based on the frame *f* + 1 which they belong to. Using the radius of the “one” event in frame *f* as threshold, we perform the same agglomerative merging as in the multiple-to-one situation above. If all the events in the “multiple” are merged together, the matching reduces to one-to-one. If not, the multiple events after merging are considered as newly appeared cells and assigned with new unique cell IDs.

### Backward matching of consecutive frames

During the forward matching process, when events are merged by agglomerative clustering in the multiple-to-one or one-to-multiple matchings, the merged events are assigned with new cell IDs. When there is a one-to-multiple matching, the multiple events in the next frame receive new cell IDs. Those events with new cell IDs are not associated to any events in the previous frames, but their corresponding cells may exist in the previous frames. The backward matching addresses this issue.

The backward matching algorithm is almost identical to the forward matching algorithm, except for two key differences. First, the backward matching process starts from the last frame, and goes back in time to match events in the current frame to events in its previous frame. Second, when performing the agglomerative merging in multiple-to-one or one-to-multiple matchings, the pairwise matrix of maximum distances between the multiple events is computed based on their associated cells in all frames.

**Fig 6:**
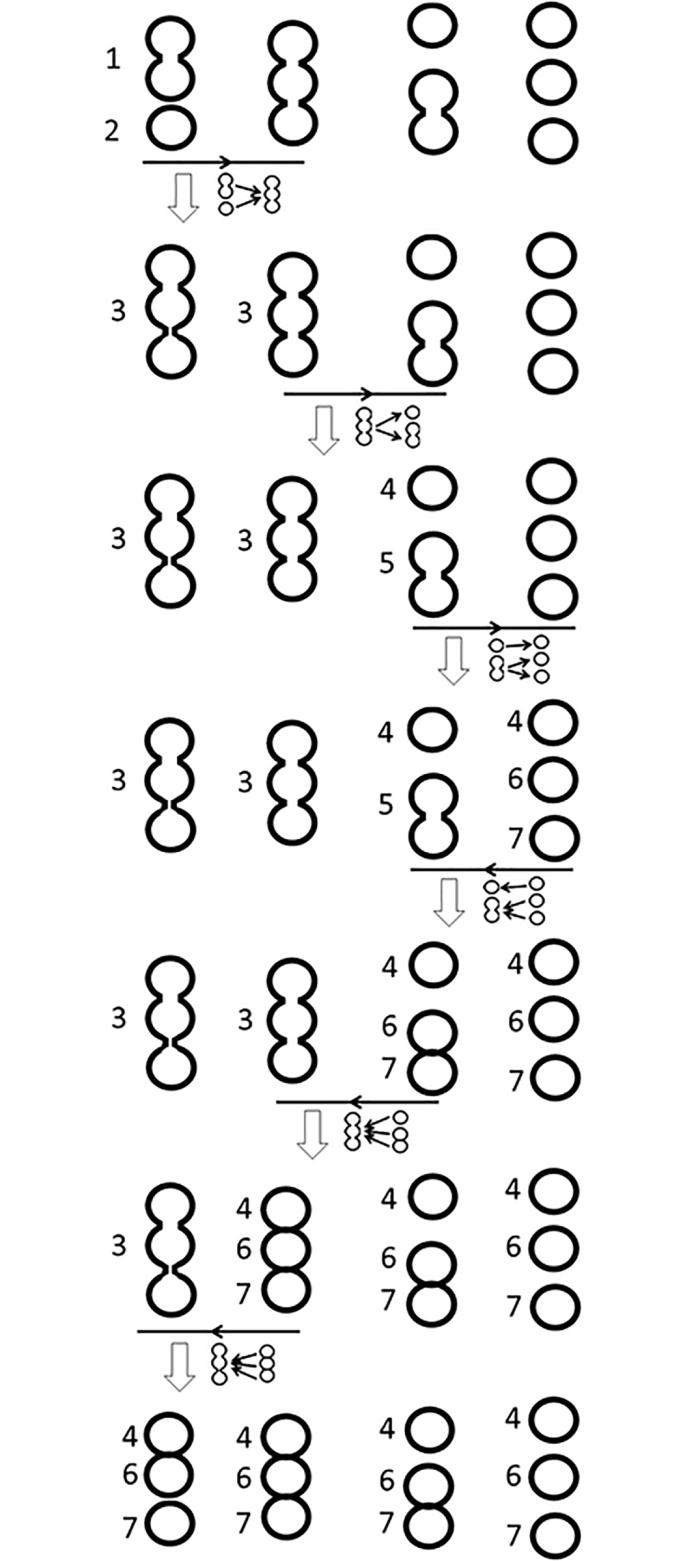
The forward and backward matching process. Each column corresponds to one image frame. Left-to-right is the forward directions. Numbers are used to indicate the cell ID associated to each event. Each vertical arrow represents one step of the forward or backward matching between two consecutive frames. As the algorithm proceeds, multiple-to-one matchings either cause the multiple merge or the one to split. One-to-multiple matchings either cause the multiple to merge (not contained in this example), or cause the multiple to receive new cell IDs.

The combination of forward and backward matching enables accurate tracking of cells involved in complex sequences of collisions and detachments. One illustrative example is shown in Fig 6. Assume we have a short video of four frames, and first row of Fig 6 represents the events obtained from foreground identification and segmentation. The number of events in these four frames are two, one, two, and three. Upon initialization of the algorithm, the two events in the first frame receive unique cell IDs (1, 2). Each subsequent row shows the tracking result after one step of the forward and backward matching. From the first to the second frame, the best matching is two-to-one. Since the two events in the first frame are not well separated compared to the size of the one event in the second frame, they are merged into one event and assigned with a new cell ID (3). After the first iteration of forward matching, there is only one event in the first frame. The matching from the second to the third frame is one-to-two, and the two events in the third frame receive new cell IDs (4, 5) because they are decently separated compared to the size of the one event in the second frame. Similarly, the matching from the third and fourth frame is also one-to-two with decent separation between the two, and therefore, new cell IDs (6, 7) are assigned to events in the fourth frame. The backward matching from the fourth to third frame has a two-to-one matching. Since the two (6, 7) are decently separated, the one (5) is split into two events and assigned with the corresponding cell IDs. The matching from the third and second frame is three-to-one. Since the three events (cells 4, 6, 7) have been well separated in other frames, the one (3) is split to three events and assigned with appropriate cell IDs. The last step of backward matching is also three-to-one, and follows the same operation. At the end of the forward and backward matching process, all frames have three events which are correctly associated to three cell IDs. Performance of the matching algorithm can be affected by the quality and availability of the data. In the above example, if the fourth frame does not exist, the algorithm will only be able to identify two cells, one is the upper cell (4), and the other is the doublet (5) of the middle and bottom cells in the third frame.

## Results

We collected 14 recordings under two perturbation conditions (data available in S1 File). The recordings under 0CD condition contained 23312 frames and 23416 events in total, indicating that the 0CD recordings were sparse, and each frame contained only one cell on average. In fact, 36% of the frames were empty, 38% of the frames contained one cell, and 24% of the frames contained 2 or more cells. This was because K562 cells under 0CD condition were stiff and easily slowed down by the ridges, and thus, they tended to clog the microfluidic channel if the cell concentration was higher and more cells were passing through the channel simultaneously. The cell concentration under the 1.5CD condition was higher. The recordings under 1.5CD condition contained 9718 frames with 27026 events, translating to roughly 3 events per frame.

**Fig 7:**
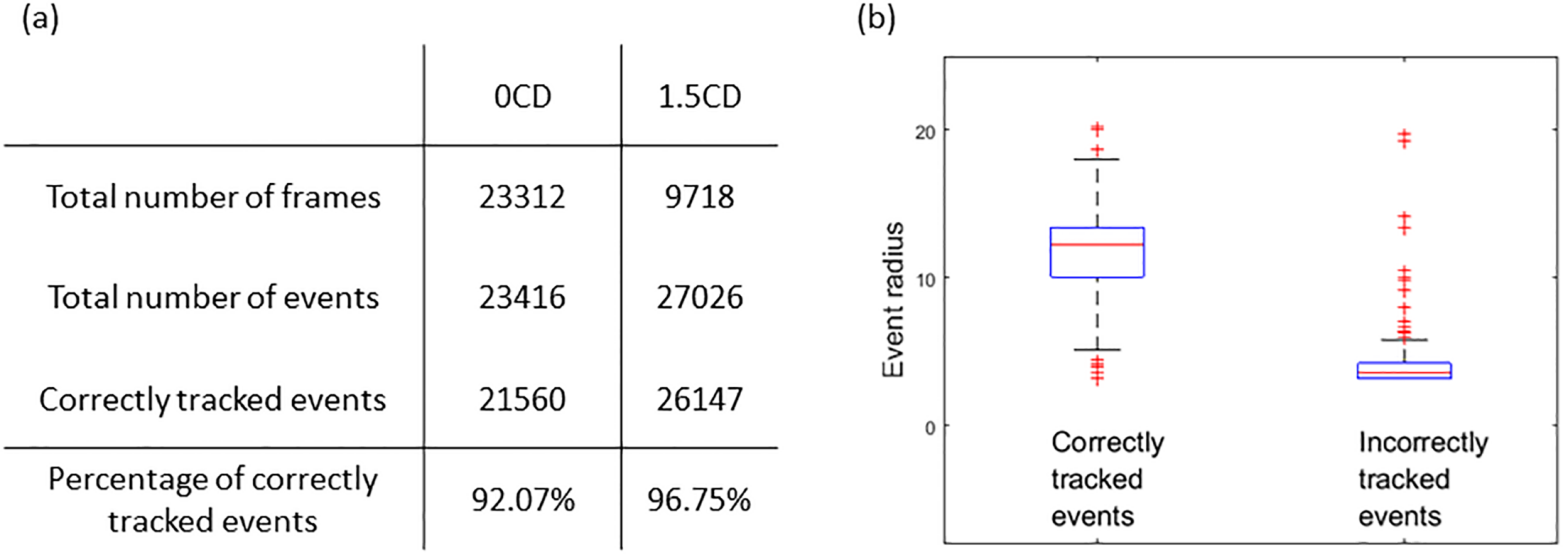
Summary of tracking results on recordings of cells under two perturbation conditions. (a) Size of the data and tracking performance. (b) Comparing the size of events associated to correct and incorrect trajectories, the incorrectly tracked events are mostly small debris.

The forward and backward tracking process associated the events to cells and trajectories. Trajectories that started and ended at the boundaries of the field of view were considered as correctly tracked trajectories, whereas incorrectly tracked trajectories were those either started or ended in the middle of the field of view. Under the two perturbation conditions, the percentage of events associated to correctly tracked trajectories were 92% and 97% respectively. Fig 7 shows that events associated to incorrectly tracked trajectories were much smaller compared to the correctly tracked cells. Those incorrectly tracked events were typically small debris that had low contrast and high speed when moving through the device.

**Fig 8:**
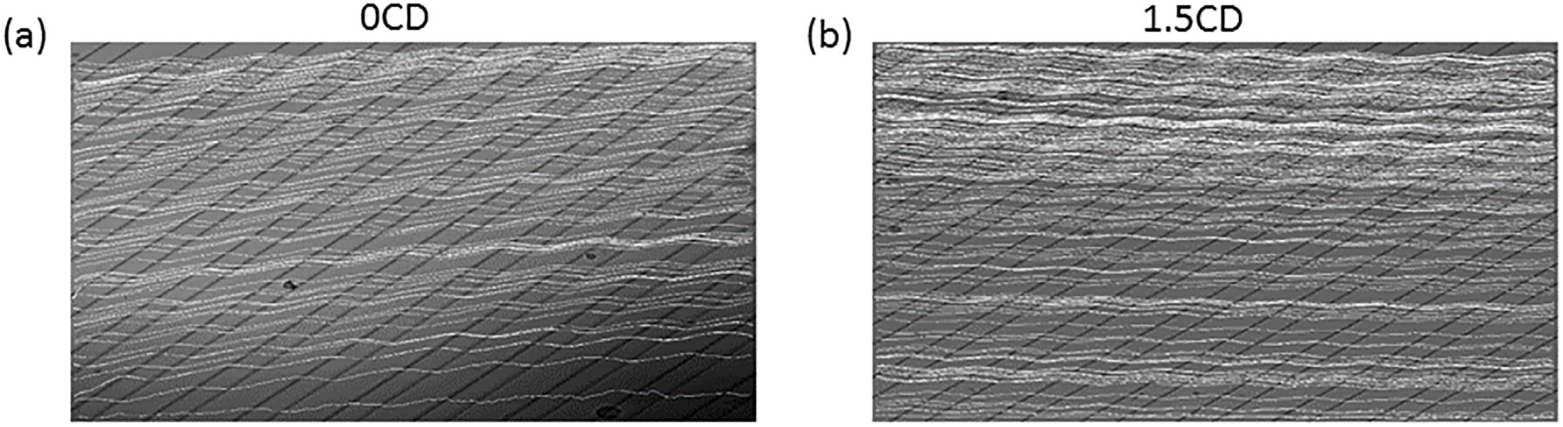
Visualization of tracking results. Overlay of the correctly tracked trajectories on the background of the recordings.

**Fig 9:**
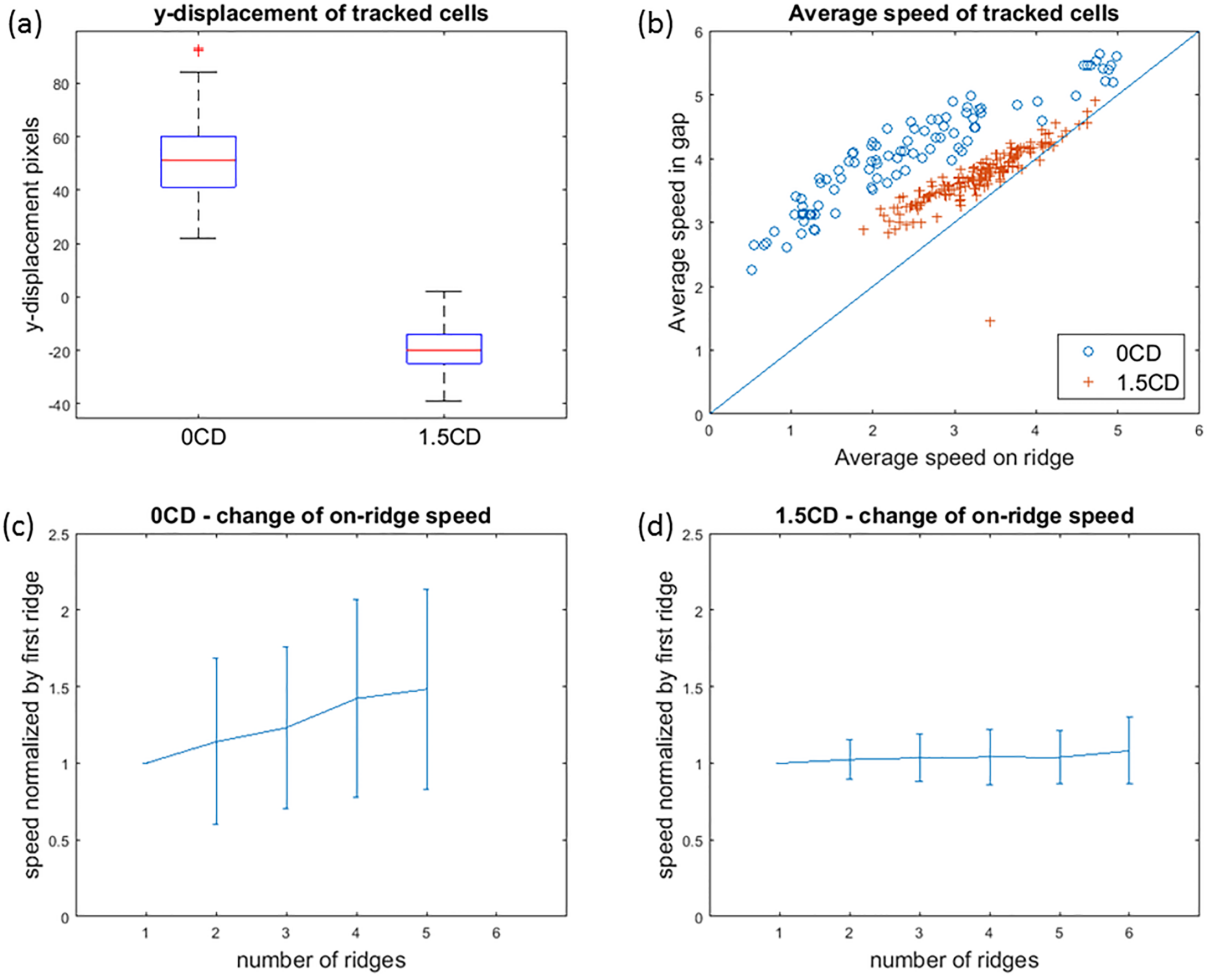
Comparison of 0CD (stiff) and 1.5CD (soft) cells. (a) Stiff cells drift up along the y-direction, whereas soft cells tend to have a slightly negative drift. (b) The speed on ridge of stiff cells is smaller than their speed in gap. Soft cells are less affected by the ridges. (c) Stiff cells tend to travel faster after passing each ridge, whereas (d) soft cells travel at a relatively constant speed irrespective of the ridges.

Fig 8 shows overlays of the correctly tracked trajectories. We can see that cells under 0CD condition drifted up along the y-direction, whereas cells under 1.5CD condition exhibited a slightly negative drift along the y-direction. This was a significant difference as shown in Fig 9a.

For each cell/trajectory, we computed its average speed when overcoming the ridges, and its average speed when traveling in the gaps between the ridges. The results were visualized in Fig 9b. Cells under 1.5CD condition scatter close to the 45 degree line, meaning that their speed on ridges and speed in gaps are similar, because they were soft and could easily deform and overcome the ridges. The variation of speed was tightly correlated with size, with smaller cells traveling faster and large ones traveling slower. Under 0CD condition, cells were stiff and more affected by the ridges, and thus, their speed on ridges was much slower then their speed in gaps. The two populations in Fig 9b can be well-fitted by two straight lines, suggesting that the stiffness of cells was relatively constant within each population, and the slopes were useful for quantifying the stiffness.

The trajectories enabled quantification of subtle changes of cells as they overcame several ridges. For each trajectory, we computed the cell’s average speed when passing each ridge, and normalized by its average speed on the first ridge. For cells under 0CD condition, the mean and standard deviation of the normalized average speed per ridge was shown in Fig 9c. We observed that stiff cells tended to travel slightly faster as they passed more ridges. Given the large standard deviation shown in Fig 9c, this trend of increasing speed was not statistically significant. However, it was consistent with previous observations that stiff cells can become softer after repeated biomechanical perturbation. In contrast, Fig 9d showed that the soft cells maintained constant speed with respect to the number of ridges they encountered.

## Conclusions

We developed a computational algorithm to automatically and accurately extract the cell trajectories in high-speed video recordings of microfluidic cell sorting devices. We tested the algorithm on recordings of K562 cells under two perturbation conditions, representing stiff and soft cells. The algorithm successfully handled the collision and detachment of cells. We showed that the automatically extracted trajectories correctly captured the difference in stiffness between the two perturbation conditions. The tracked trajectories revealed the subtle increase in speed when the stiff cells pass through consecutive ridges, which is an indication that cell biomechanical properties may change when passing through the ridges. The accurately tracked trajectories enables future efforts of optimizing device design, for the purposes of modeling and quantification of the dynamical changes of cell biomechanical properties in the context of microfluidics.

## Supporting information

S1 File Source code and data. This file contains the source code of the cell tracking algorithm, video recordings collected in the microfluidic experiments, and example use cases.
(ZIP)
